# Shared Genetics between Depression and Cardiometabolic Disorders

**DOI:** 10.1192/j.eurpsy.2025.1533

**Published:** 2025-08-26

**Authors:** P. S. Kundi, M. Syed, B. A. Syed, D. Sahota

**Affiliations:** 1 Vancouver Coastal Health, Vancouver; 2 Psychiatry, Peace Arch Hospital, White Rock, Canada; 3 Oregon State University, Corvalis, United States; 4 McMaster University, Hamilton, Canada

## Abstract

**Introduction:**

Several studies have discovered associations between depression and cardiovascular disease risk factors and patients with Coronary Artery Disease with comorbid Depression have worse prognosis. There is growing evidence of polygenic overlap between depression, Coronary Artery Disease and other Cardiovascular risk factors and may suggest molecular mechanisms underlying the association between depression and raised cardiovascular disease risk.

**Objectives:**

Depression and cardiometabolic disorders are both heritable and both are caused by a mix of genetic and environmental factors. Genetic factors contribute to 31–42 % in MDD (Sullivan et al. 2000 ), 30–60 % in coronary artery diseases (Marenberg et al. 1994 ), 26–69 % in type 2 diabetes (Almgren et al. 2011 ; Poulsen et al. 1999 ), 24–37 % in blood pressure (hypertension) (Van Rijn et al. 2007 ), 35–48 % in heart rate variability (Kupper et al. 2004 ), 40–70 % in obesity (body mass index) (Willyard 2014 ), and 58–66 % in the level of serum lipids (Knoblauch et al. 1997 ). Moreover, there are fairly high genetic co-heritabilities (genetic correlations) between depression and the different cardiometabolic disorders suggesting the influence of pleiotropic genes and shared biological pathways within them.

**Methods:**

Literature search was conducted and appropriate material was then extracted to examine the hypothesis.

**Results:**

Identification of shared molecular pathways supports a growing evidence base for cross-diagnostic treatment. Besides, further exploration of overlapping molecular pathophysiology can unveil novel targets for drug development and repurposing of existing medications. Also, cardiometabolic disorders can increase the risk of poor response to standard treatments in mood disorders. Lastly, studying shared pathways of depression and somatic disorders can untangle the clinical and genetic heterogeneity that underlies in these illnesses.

**Conclusions:**

Genetic studies have suggested the involvement of pleiotropic genes in the comorbidity between depression and cardiometabolic disorders. While our abstract attempts to provide some insight into the common mechanisms and role of pleiotropic genes, in-depth understanding of how these genes mediate the association between depression and cardiometabolic diseases requires future larger scale comprehensive cross-disorder research. This will enable us to better understand why patients suffer from multiple diseases at a time and how multi-morbidities influence pharmacological treatment response to diseases.

**Disclosure of Interest:**

None Declared

